# Time-of-flight electron energy loss spectroscopy using TM_110_ deflection cavities

**DOI:** 10.1063/1.4962698

**Published:** 2016-09-13

**Authors:** W. Verhoeven, J. F. M. van Rens, M. A. W. van Ninhuijs, W. F. Toonen, E. R. Kieft, P. H. A. Mutsaers, O. J. Luiten

**Affiliations:** 1 Department of Applied Physics, Coherence and Quantum Technology Group, Eindhoven University of Technology, P.O. Box 513, 5600 MB Eindhoven, The Netherlands; 2 FEI Company, Achtseweg Noord 5, 5651 GG Eindhoven, The Netherlands

## Abstract

We demonstrate the use of two TM_110_ resonant cavities to generate ultrashort electron pulses and subsequently measure electron energy losses in a time-of-flight type of setup. The method utilizes two synchronized microwave cavities separated by a drift space of 1.45 m. The setup has an energy resolution of 12 ± 2 eV FWHM at 30 keV, with an upper limit for the temporal resolution of 2.7 ± 0.4 ps. Both the time and energy resolution are currently limited by the brightness of the tungsten filament electron gun used. Through simulations, it is shown that an energy resolution of 0.95 eV and a temporal resolution of 110 fs can be achieved using an electron gun with a higher brightness. With this, a new method is provided for time-resolved electron spectroscopy without the need for elaborate laser setups or expensive magnetic spectrometers.

## I. INTRODUCTION

The generation of ultrashort electron pulses has allowed for the investigation of processes on atomic length scales and femtosecond time scales.^1–3^ A widely used technique is to generate electron pulses through photoemission from a cathode using a femtosecond laser. With this, both diffraction and direct imaging can be performed. Recently, the use of femtosecond electron pulses for electron energy loss spectroscopy (EELS) in a pump-probe setup was demonstrated by Carbone *et al.*,^4^ providing yet another powerful method to extract time-resolved information from a specimen.

The common approach to resolve electron energies is to use sector magnets or Wien filters. Since these give small energy-dependent deflections, typically a few *μ*m per eV,^5^ they often require significant spatial filtering through slits. However, for ultrashort pulses, both the current and transverse coherence are limited, making these methods difficult to use for time-resolved measurements. Therefore, time-of-flight (ToF) methods provide an interesting alternative, in which energies can be measured without spatial filtering. Recently, Gliserin *et al.*^6^ demonstrated the use of a drift tube, in which electrons are slowed down almost to a complete stop, causing differences in velocity to translate into large differences in drift time. These drift times can then be measured using a micro-channel plate detector connected to a time-to-digital converter. With this, a high resolution can be achieved with a relatively large current throughput.

An alternative approach to generate ultrashort electron pulses is to sweep a continuous beam over a slit.^7,8^ Recently, it has been demonstrated that an RF cavity oscillating in the TM_110_ mode can be used to chop a continuous beam into ultrashort pulses without a significant loss of emittance by focusing the electron beam in the center of the cavity.^9^ Using state-of-the-art synchronization schemes,^10,11^ these pulses can then be accurately timed with a femtosecond laser, allowing for pump-probe measurements to be performed with a good spatial and temporal resolution.

It has also been proposed that the time-dependent deflection of a TM_110_ cavity can be used to measure differences in electron flight times.^9^ These streak cavities have been used before for bunch length diagnostics,^12^ and they provide a high temporal resolution. This makes them suitable ToF spectrometers.

In this paper, the feasibility of using TM_110_ cavities for the generation of pulses and the measurement of electron flight times is demonstrated. The resolution of the setup presented here is currently limited by the brightness of the tungsten filament electron gun used. Through simulations, it is shown that both the energy and time resolution can be improved using a high brightness gun.

An interesting advantage of this method is that the energy spectrum can be imaged along one direction simultaneously with a diffraction pattern along the other direction, allowing for the dispersion curve of fundamental excitations to be measured with a high temporal resolution.

## II. PRINCIPLE

**FIG. 1.**
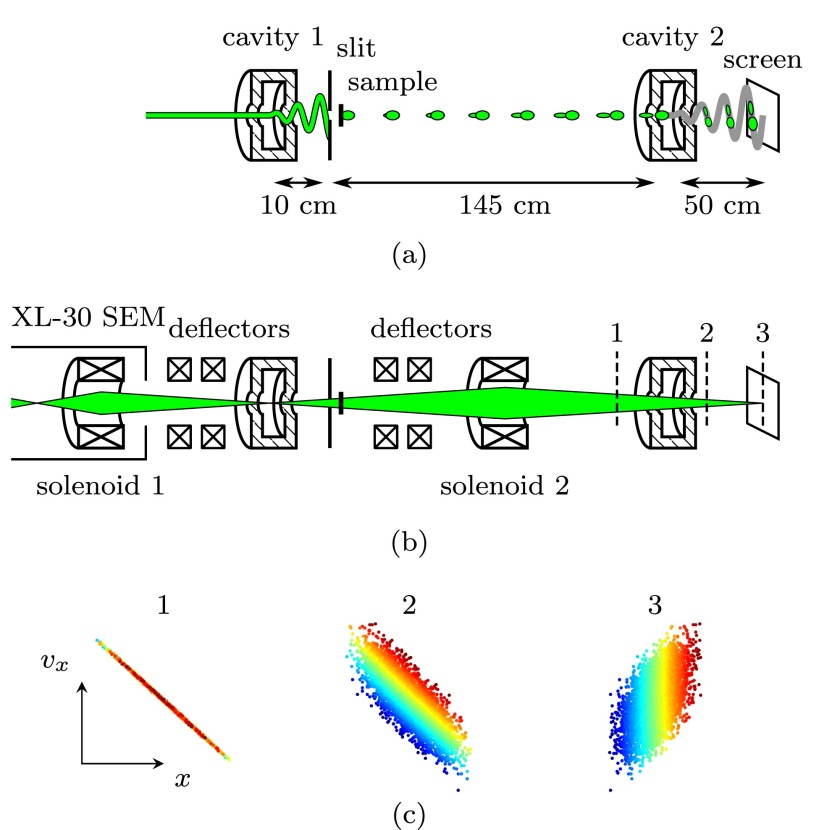
(a) General principle of a ToF-EELS measurement using two cavities. Pulses are generated by a first cavity, after which they interact with a sample. Electrons with different energies are then separated temporally in the drift space, after which the temporal distribution is streaked across the detector by the second cavity. (b) Electron-optical elements added to the setup, together with the transverse envelope of the beam. Dashed lines indicate the positions of the (c) phase space diagrams. Shown in these diagrams is the relative transverse position and velocity of particles in a converging beam moving through the cavity and onto the detector, with the color coding corresponding to the arrival time at the second cavity. Within the crossover of the beam, the different arrival times are imaged onto the transverse position.

Figure 1(a) schematically depicts the principle behind the ToF measurement. Electron pulses are first created by sweeping a continuous beam over a slit using a TM_110_ cavity. The pulsed beam then interacts with a sample, thereby acquiring a specific energy loss distribution which translates into a specific velocity distribution. After propagating through a drift space, this will cause the electrons to arrive at the second cavity at different times. The time-dependent deflection of this cavity will then separate these electrons onto the detector, allowing for the differences in arrival time to be measured.

From these arrival times, it is straightforward to calculate the corresponding energies. For small differences compared to the total kinetic energy, this energy difference can be approximated by

$$\frac{\Delta E}{E_{0}}\approx (\gamma_{0}+\gamma_{0}^{2})\frac{\Delta v}{v_{0}}\approx -(\gamma_{0}+\gamma_{0}^{2})\frac{\Delta t}{t_{0}},$$ (1)

with Δ*E* the energy difference, Δ*v* the corresponding difference in velocity, and Δ*t* the difference in arrival time. In this equation, $E_{0}=mc^{2}(\gamma_{0}-1)$ is the kinetic energy, $\gamma_{0}={\left(1-v_{0}^{2}/c^{2}\right)}^{-1/2}$ is the Lorentz factor, and *v*_0_ and *t*_0_ are the unperturbed velocity and flight time of the electrons, respectively.

From Equation (1), it can be seen that the resolution is limited by the initial temporal spread of the pulse. As an example, for a 30 keV electron beam, it takes approximately 14.7 ns for the electrons to traverse the 1.45 m drift space that is used in our setup. When pulses are created with a length of 100 fs, the instrumental resolution is expected to be 0.45 eV.

However, care must be taken that the energy spectrum is imaged on the detector as accurately as possible, which can be accomplished by adding two crossovers to the setup. The first one has to be placed at the center of the first cavity to minimize emittance growth.^9^ The second one is placed at the detector, as is explained below. In addition to the two cavities, this scheme therefore also requires two charged particle lenses. Figure 1(b) shows all the electron-optical elements used in the setup, together with the beam envelope when the cavities are turned off.

Figure 1(c) explains the principle of imaging the arrival times using a lens and a cavity in more detail. Shown here are transverse *x–v_x_* phase space diagrams of the electrons at the positions indicated by the three dashed lines in Fig. 1(b), with the color coding corresponding to different arrival times at the second cavity. Before entering this cavity, there is a linear correlation between the position and velocity since the beam is converging. In addition to this converging motion, electrons gain a transverse velocity depending on their arrival time when traversing the cavity. Within the crossover of the beam, each position on the detector will then correspond to a particular arrival time at the second cavity, which can be seen from the correlation between the arrival times and the transverse positions in the third diagram. The combination of an electron-optical lens and a cavity therefore allows for an accurate image to be made of the arrival times, and thus the energy spectrum.

Since a crossover is required at the center of the first cavity, the beam diverges towards the slit. Because of this, electrons from a larger range of deflection angles can pass through the slit, increasing the temporal length of the pulses. Assuming a uniform distribution of the beam and non-relativistic speeds, the pulse length *τ* for a beam with a finite beam width *w* at the position of the slit is given by^13^

$$\tau =\frac{m_{e}\left(s+w\right)}{2|q_{e}|lB_{0}},$$ (2)

with *s* the slit size, *l* the distance to the slit, *B*_0_ the magnetic field amplitude, and *m_e_* and *q_e_* the electron mass and charge. In order to generate short pulses with minimal emittance growth, it is therefore important to focus the beam inside the first cavity with a small divergence angle, thus keeping the beam width *w* small.

## III. EXPERIMENTAL SETUP

In our setup, a continuous 30 keV electron beam is generated using a Philips XL-30 SEM with a tungsten filament gun. The condenser lens of the microscope is used to control the current used for the experiment. Since no imaging is done in this setup, the final lens of the microscope can be used to focus the beam at the center of the first cavity. An additional solenoid lens is used to refocus the beam on a TVIPS TemCam-F216 detector.^14^ In order to steer the beam through both cavities, two sets of deflection coils are added.

Both for the generation of electron pulses and for the measurement of the flight time, TM_110_ streak cavities are used. By loading the cavities with ZrTiO_4_, a dielectric material with a high permittivity and a low loss tangent, both the size and the power consumption can be drastically reduced.^13^ The cavities are driven by a Mini-Circuits ZX95–3060C+ Voltage Controlled Oscillator (VCO) at a frequency of 2.9985 GHz. The signal of the VCO is split up and sent through two Mini-Circuits ZHL-16W-43+ power amplifiers. The RF phase difference between the two cavities can be adjusted using a phase shifter placed before one of the amplifiers. At an input power of 10 W, the magnetic field amplitude is *B*_0_ = 1.2 ± 0.1 mT.

A slit with a width of *s* = 10 *μ*m is placed at a distance *l* = 10 ± 1 cm behind the first cavity, and samples can be inserted immediately behind the slit. The drift space between the slit and the second cavity is 145 ± 1 cm, and the distance from the second cavity to the detector is 50 ± 1 cm.

**FIG. 2.**
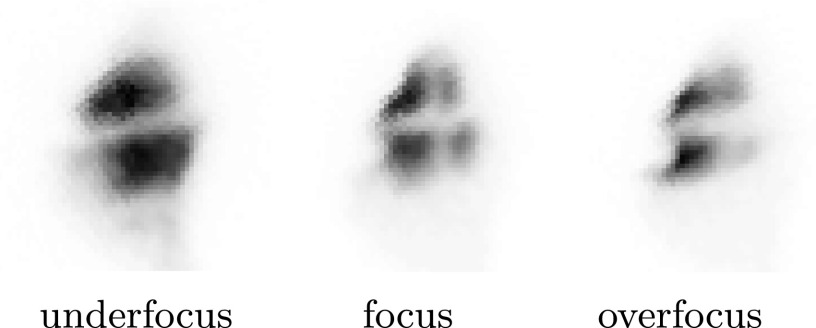
Inverted contrast images of a TEM grid on the detector using a pulsed beam for different focal strengths of the SEM final lens. Focal strength of the final lens increases from left to right.

To establish that the emittance is minimized after the first cavity, an image of the sample is made with a pulsed electron beam. The resolution can then be optimized in the sweeping direction by adjusting the focal position. Figure 2 shows this principle when two crossing copper lines on a TEM grid are imaged. The electrons are streaked in the horizontal direction by the first cavity, as can be seen from the growth of emittance in this direction in the case of under- and overfocus, causing the vertical line to slowly disappear at larger defocus.

Using two phosphor screens, the semi-angle of the beam is measured to be *α* = 0.41 ± 0.06 mrad, so that the beam width is w=2lα=(8±2)·10 μm. The pulse length given by Equation (2) is then *τ* = 2.1 ± 0.5 ps. Using Equation (1), this gives an instrumental resolution of 9 ± 2 eV in theory.

## IV. RESULTS

**FIG. 3.**
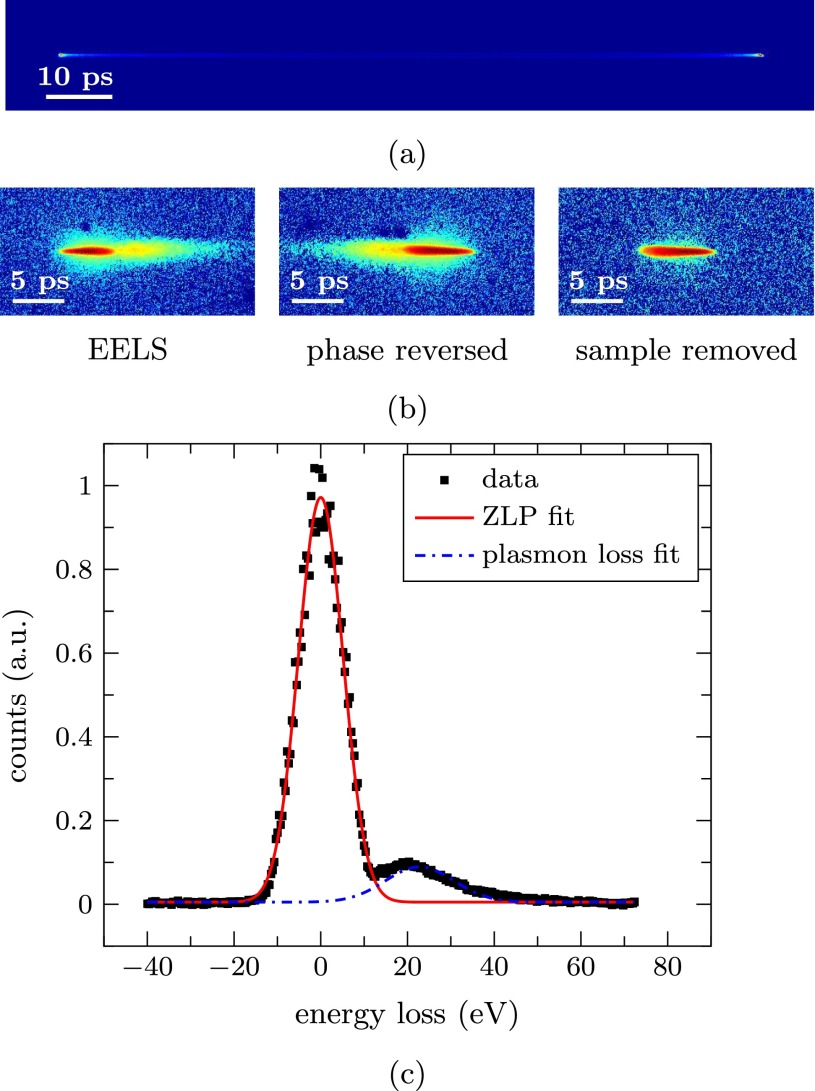
(a) Image of the full streak on the detector. (b) Detector signal color coded on a logarithmic scale, showing from left to right the EELS measurement, the same measurement with the streaking direction of the second cavity reversed, and a measurement without a sample. (c) The energy loss spectrum acquired from the left image of (b), together with fits of the ZLP and the plasmon loss peak.

In order to demonstrate the feasibility of our ToF-spectrometer, a typical cross grating sample,^15^ containing a polycrystalline gold layer deposited on a carbon film, has been inserted into the setup. The measurements on this sample are shown in Fig. 3.

Figure 3(a) shows the full streak on the detector made with a continuous beam. Since the period of the cavity is known, the length of the streak is used to calibrate the arrival times. Figure 3(b) shows the signal on the detector for a pulsed beam, color coded on a logarithmic scale. Shown here from left to right are a measurement with a sample inserted, the same measurement after the streaking direction of the second cavity has been reversed using the phase shifter, and a measurement without the sample. This shows a clear signal arising from electrons that have lost energy to the sample, with the direction of the tail corresponding to later arrival times.

Figure 3(c) shows the spectrum acquired by summing over the detector pixels in the direction transverse to the streak, plotted against the energies that have been calculated from the arrival times. The solid red curve is a fit to the zero-loss peak (ZLP), and the dashed-dotted blue curve is a fit to the peak at the plasmon resonance, at a loss of approximately 20 eV. From the ZLP, we find an energy resolution of $\Delta E_{\text{FWHM}}=12\pm 2\;\text{eV}$. The corresponding spread in arrival times is $\Delta t_{\text{FWHM}}=2.7\pm 0.4\;\text{ps}$. This spread is an upper limit to the temporal resolution for the setup, since it is measured at the end of the setup, and not directly at the sample, therefore including broadening of the pulse throughout the drift space.

Note that the ZLP width is different for the two RF phases in Fig. 3(b). This can be attributed to a misaligned solenoid lens, which gives rise to *t–x* correlations at the entrance of the second cavity that either assist or oppose the streak, depending on the streaking direction. Since this is difficult to account for during post-processing, more care has to be taken in future measurements for the alignment of the solenoid.

## V. CHARGED PARTICLE TRACKING SIMULATIONS

In order to investigate the limits of the proposed technique, charged particle tracking simulations have been performed using General Particle Tracer (GPT) code.^16^ Cavities are modeled using realistic fields, including fringe fields. First, the limiting factors of the present setup are investigated.

The divergence of the electron beam was determined to be 0.41 ± 0.06 mrad when a crossover is placed at the center of the first cavity. The normalized emittance of this setup has been determined previously to be 9 × 10^−10^ mrad.^13^ Typically, a tungsten hairpin emitter has an energy spread of $\Delta E_{\text{FWHM}}=1.5–3\;\text{eV}$.^17^ In the simulations, the energy spread of the gun is assumed to be 2 eV, and a Gaussian beam profile is taken for the continuous beam coming from the microscope.

The continuous beam is then swept over the slit in the simulations. With these numbers, pulses are found with a pulse length of $\Delta t_{\text{FWHM}}=2.09\;\text{ps}$, and an energy spread of $\Delta E_{\text{FWHM}}=4.63\;\text{eV}$. This increase in energy spread is caused by the nonuniform off-axis electric fields inside the cavity. Energy loss can then be added to the pulse, after which the rest of the setup is simulated. From the width of the ZLP on the detector, an energy resolution of 10.8 eV is then found, close to the experimental value. The resolution is therefore not only limited by the large pulse length but also the increase in energy spread due to the cavity.

However, both can be decreased by using an electron gun with a lower emittance, allowing for the divergence angle to be decreased, which results in a smaller pulse length. Furthermore, with a lower emittance, the spread in off-axis electric fields probed by the beam is smaller, decreasing the energy spread induced by the first cavity.

To prove this, simulations have been run using the parameters of a field emission gun (FEG). These guns typically have an energy spread of $\Delta E_{\text{FWHM}}=0.2-0.7\;\text{eV}$,^17^ and a normalized emittance at a given current of up to two orders of magnitude smaller than a tungsten hairpin emitter. To get an optimal pulse length, the divergence should be decreased as far as allowed by the finite emittance. The slit size should then be chosen to be equal to the beam size at the slit. A smaller slit would decrease the pulse length, but at the cost of current in the pulse. It is therefore favourable to increase the magnetic field strength, as can be seen from Equation (2).

In the simulations, this is done by placing a 10 *μ*m aperture 10 cm in front of the cavity. This gives good results, while still being experimentally feasible. The beam is then swept over a 10 *μ*m slit by the first cavity. The FEG is assumed to have an energy spread of 0.5 eV and a normalized emittance of 3 × 10^−12^ m rad.

**FIG. 4.**
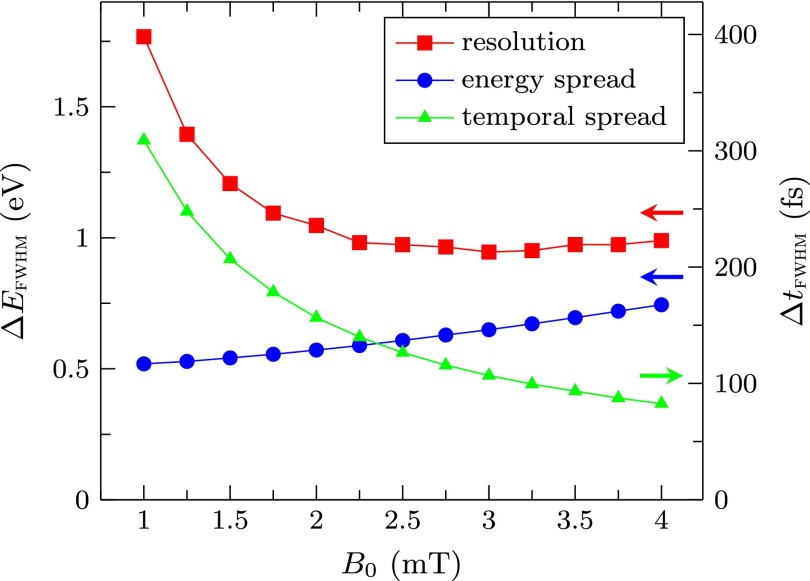
Simulated energy resolution of the setup, together with the energy spread and the temporal spread of the pulses for different field strengths inside the first cavity. Left and right axes are linked together through Equation (1).

Figure 4 shows the resolution of the setup for different field strengths in the first cavity, together with the energy spread and temporal spread of the pulses created at the slit. The left and right *y*-axes are linked together through Equation (1). From this figure, it can be seen that an increase in the magnetic field amplitude decreases the pulse length, but also increases the energy spread due to an increase in the off-axis electric fields inside the cavity. Because of this, we find a minimum energy resolution of 0.95 eV at a field of 3 mT. The temporal resolution is found to be 110 fs at this field strength.

## VI. SUMMARY AND CONCLUSIONS

We have shown that a pair of pillbox cavities oscillating in the TM_110_ mode can be used for time-resolved EELS measurements. In the first proof-of-principle measurement, a temporal resolution of 2.7 ± 0.4 ps combined with an energy resolution of 12 ± 2 eV is obtained at an energy of 30 keV. Simulations show that a temporal resolution of 110 fs and an energy resolution of 0.95 eV can be achieved when using a source with a lower emittance such as an FEG. The important benefits of this setup are that it does not require femtosecond lasers to generate electron pulses by photoemission, and that measurements can be done without loss of current. In the future, the setup will be adjusted so that an energy spectrum can be measured in one direction with a diffraction pattern in the other direction, allowing for both the transfer of energy and momentum to be measured simultaneously, and thus the dispersion curve of fundamental excitations. This provides a useful and versatile new tool for time-resolved spectroscopy.
